# Life Lost Due to Premature Deaths in New South Wales, Australia

**DOI:** 10.3390/ijerph6010108

**Published:** 2009-01-06

**Authors:** Daminda P. Weerasinghe, Farhat Yusuf, Nicholas J. Parr

**Affiliations:** 1 Department of Cardio Thoracic Surgery, Prince of Wales Hospital, Randwick, NSW, Australia; 2 Faculty of Business and Economics, Macquarie University, North Ryde, NSW, Australia; E-Mails: f.yusuf@efs.mq.edu.au (F. Y.); nparr@efs.mq.edu.au (N. J. P.)

**Keywords:** Potential life lost, premature mortality, Australia

## Abstract

This study attempts to measure premature mortality, in addition to overall death rates, in order to provide more information that can be used to develop and monitor health programmes that are aimed at reducing premature (often preventable) mortality in New South Wales (NSW), Australia. Premature years of potential life lost (PYPLL) and valued years of potential life lost methods are applied for mortality data in NSW from 1990 to 2002. Variations in these measures for 2001 are studied further in terms of age, sex, urban/rural residence, and socio-economic status. PYPLL rates for all leading causes of death have declined. It is shown that the average male to female ratio of PYPLLs is highest for accidents, injury and poisoning (3.4:1) followed by mental disorders (2.7:1) and cardiovascular diseases (2.6:1). Although fewer women than men die of cardiovascular diseases, there is a greater proportionate importance of cerebrovascular mortality among women. In order to further reduce premature deaths, programs are required to improve the health of people living in lower socio-economic status areas, especially in rural NSW. Targeted regional or community level programs are required to reduce avoidable deaths due to accidents, injury and poisoning occasioned by motor vehicle accidents, poisoning and suicide among young adults.

## 1. Introduction

General mortality rates in a population tend not to reflect mortality trends of young persons, as they are dominated by chronic diseases among the elderly, especially diseases of the cardiovascular system and malignant neoplasms. Measuring premature mortality in addition to overall death rates provides more information that can be used both to develop and to monitor health programmes that are aimed at reducing premature (and often preventable) mortality.

‘Years of potential life lost’ (YPLL) refers to the number of years of potential life not lived when a person dies ‘prematurely’ [1]. Premature death refers to a loss of years of productive life due to death before a selected cut-off age. Debate persists over the definition of ‘premature’ death, but the majority of studies use YPLL-65, which defines premature death as death before age 65. This measure is also referred as premature years of potential life lost (PYPLL). In the computation of ‘valued years of potential life lost’ (VYPLL), the lifespan of each individual is divided into the following three groups: investment years (birth to age 15), production years (age 15 to 64) and consuming years (age 65 years and over). This is known as the investment-producer-consumer model (IPC model) [2].

The aim of this article is to use potential life lost measures - i.e. to calculate YPLL, PYPLL and VYPLL - to examine the relative impact on the population of New South Wales (NSW) of premature deaths due to a selected group of broad causes. The respective formulae for these measures are presented in Appendix 1. The death data of NSW residents over a period from 1990 to 2002 are analysed for the following groups of causes of death; infectious and parasitic diseases; malignant neoplasms; mental disorders; diseases of the cardiovascular system; diseases of the respiratory system; diseases of the digestive system; and accidents, injury and poisoning. The research questions addressed in this study are to identify the differentials in PYPLL due to premature deaths by age, sex, urban/rural residence, and Socio-Economic Index For Areas (SEIFA) status.

## 2. Methods

The Australian Bureau of Statistics (ABS) tabulated cause of death data for the NSW resident population by age at death, gender and postcode of residence from 1990 to 2002 were obtained from the NSW Department of Health [3]. Information regarding the causes of death have been extracted from the appropriate International Classification of Diseases: ICD-9 and ICD-10 codes [4]. There could be some ICD coding limitations especially for people who died with multiple complications [5].

The crude death rates have been computed using population census data in 2001. The 2001 SEIFA of socio-economic advantage by postcode for NSW were obtained from the ABS, which have been developed with the use of census data to provide an indication of different aspects of socio-economic conditions by geographic areas [6, 7]. Principal components analysis technique was used to generate the indexes, which summarises information from a variety of social and economic variables, calculating weights that will give the best summary for the underlying variables. The index of advantage and the index of disadvantage have the broadest coverage of advantage/disadvantage, using variables that both measure and reflect advantage/disadvantage [6]. For example, a high score on the index of relative socio-economic advantage indicates that an area has certain attributes denoting “advantage”, such as a relatively high proportion of people on high incomes or a skilled workforce. Conversely, a low score on the index indicates that an area has a higher proportion of individuals on low incomes, more employees in unskilled occupations, and so forth.

The variations in PYPLL data of the selected disease groups are studied in terms of age, gender, residential area (urban/rural) and SEIFA quintiles for 2001. In order to compare the variation in trends, PYPLL data are directly standardised to December 1990 population using the formulae presented in Appendix 1. For the comparison of age and sex specific PYPLL rates, the age and sex distribution was adjusted using the same standard population.

The upper cut-off age for these measures is set at 65 years following consideration of the standard retirement age for Australia [8]. In 2001 NSW was subdivided into 4,656 suburbs and 612 distinct postal areas ([3]). The quintiles were selected by first setting all postal areas in the ascending order of the SEIFA advantage, and then equal number of postal areas were grouped into each quintile. As such the first and third quintiles consist of 123 postal areas and the second, fourth and fifth quintiles consist of 122 postal areas.

The VYPLL values are computed for 2001 for the population aged 15–64 (production period), assuming a minimum impact by the exclusion of the economically active men and women aged below 15 and above 64 years. An assumption is made in the calculation of VYPLL that every year during the production period is of equal value. The VYPLL weights for each age group computed using the 2000–02 life tables for NSW (Appendix 2). According to the IPC model, “during investment and consumer years the individual is receiving from society (negative value for society), whereas during the producer years the individual is giving back to society (positive value for society)” [2]. To explain how this model works, if a man lives to the age of 75, his net contribution to society is 25 years (−15+50−10 = +25). A man who dies at age 22.5, results in a potential net loss of 34.6 years (−15+7.5−42.5+15.4 = −34.6).

## 3. Results

In a total of 44,538 deaths recorded in NSW in 2001, nearly 80% of the persons were over 65 years at death, and 61% were over 75 years of age. The potential life lost measures computed for 2001 mortality data under different assumptions are presented in Table 1. According to 2000–02 life tables for NSW, life expectancy at birth is 77.05 years for men and 82.3 years for women.

**Figure 1.**
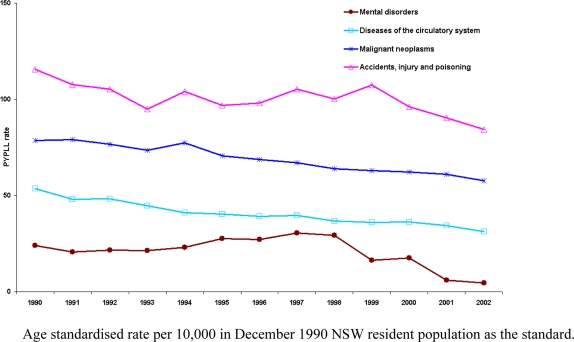
Trends in standardised PYPLL rates of leading causes of death in NSW from 1990 to 2002.

The different potential life lost measures generated for the same data show significant differences between one another, depending on the method and the cut-off age used. Over the 13 years there was a declining trend in standardised PYPLL rates (Figure 1). The average male to female ratio of PYPLLs is highest for accidents, injury and poisoning (3.4:1) followed by infectious and parasitic diseases (3.3:1), mental disorders (2.9:1), diseases of the cardiovascular system (2.6:1) and diseases of the digestive system (2.3:1). The leading causes of PYPLLs are accidents, injury and poisoning; malignant neoplasms; and diseases of the cardiovascular system, with accidents, injury and poisoning being the leading cause of PYPLL for men and malignant neoplasms for women. From 1999 to 2002, the PYPLL of accidents, injury and poisoning declined steadily for men by 18.8% and for women by 28.5%, whilst malignant neoplasms remained relatively unchanged. Although only 7.6% of male deaths and 3.6% of female deaths are recorded as being caused by accidents, injury and poisoning (mean age for male_2001_= 46.4 and for female_2001_= 60.0), the relatively high PYPLLs are due to the relatively younger age at death. Malignant neoplasms accounts for a higher number of deaths among men than among women, but the difference in PYPLLs between men and women is not great because of the higher proportion of women dying at younger ages than men.

**Figure 2.**
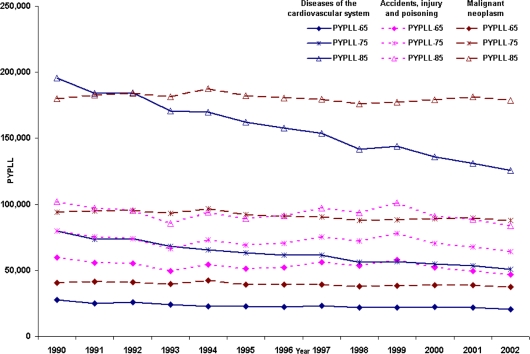
Trends in PYPLL at cut-off ages 65, 75 and 85 for diseases of the cardiovascular system, malignant neoplasms and accidents, injury and poisoning for persons in NSW from 1990 to 2002.

Long-term PYPLL trends computed with different cut-off ages (65, 75 and 85) for persons who died with diseases of the cardiovascular system, malignant neoplasms and accidents, injury and poisoning, are presented in Figure 2. These data show that, when the cut-off age increases from 65 to 75, the leading cause of PYPLL changes from accidents, injury and poisoning to malignant neoplasms. When the cut-off age is increased to 85, the difference in PYPLLs for these causes of death becomes even wider, demonstrating the fact that PYPLL measures are highly dependent on the choice of cut-off age.

The PYPLL data are further examined by dividing the deaths data by place of residence in NSW into two groups: urban and rural. Declining standardised PYPLL trends are recorded in both urban and rural areas. Over the study period, the urban to rural standardised PYPLL ratio is 0.83:1. These data indicate that people in rural areas die at younger ages than people in urban areas. When comparing the ratios applying to all of the causes of death, the urban to rural ratio of PYPLL rate is greatest for accidents, injury and poisoning (0.67:1); for diseases of the cardiovascular system, this ratio is 0.76:1.

In 2001 among NSW residents, premature deaths due to all causes led to 107,393 years of potential life lost for men and 57,203 years for women. The age distribution of PYPLL showed a bimodal curve, with the first minor peak at 20–24 years of age and the second at 50–54 years of age (Figures 3.1 and 3.2). The selected causes of death make up 81% of PYPLL for men and 71% for women. As has already been pointed out, the accidents, injury and poisoning group represents the main component of PYPLL, especially among younger men, and malignant neoplasms is the main component of PYPLL among women under 65 years of age. Diseases of the cardiovascular system are the third main contributor to PYPLL, being responsible for 21,688 years of potential life lost. In men, deaths due to accidents, injury and poisoning start increasing at school age and continue to increase until the age of 25. After age 40 there is a sharp decline. Diseases of the cardiovascular system and malignant neoplasms paint a complementary picture, with a slight increase after the age of 34 and reaching their peak at the age of 55. Similar to the situation with men, deaths due to accidents, injury and poisoning are relatively high among women in the period from school age to the age of 20, but the magnitude is far below the level of men. According to PYPLL, the most pronounced problems in women between 30 and 54 years of age are malignant neoplasms and, although far less, diseases of the cardiovascular system. The reasons for the high PYPLL at age 20–24 could be attributed to high number of deaths among men due to accidents, injury and poisoning, and at age 50–54 the number of high deaths due to malignant neoplasms among women.

**Figure 4.**
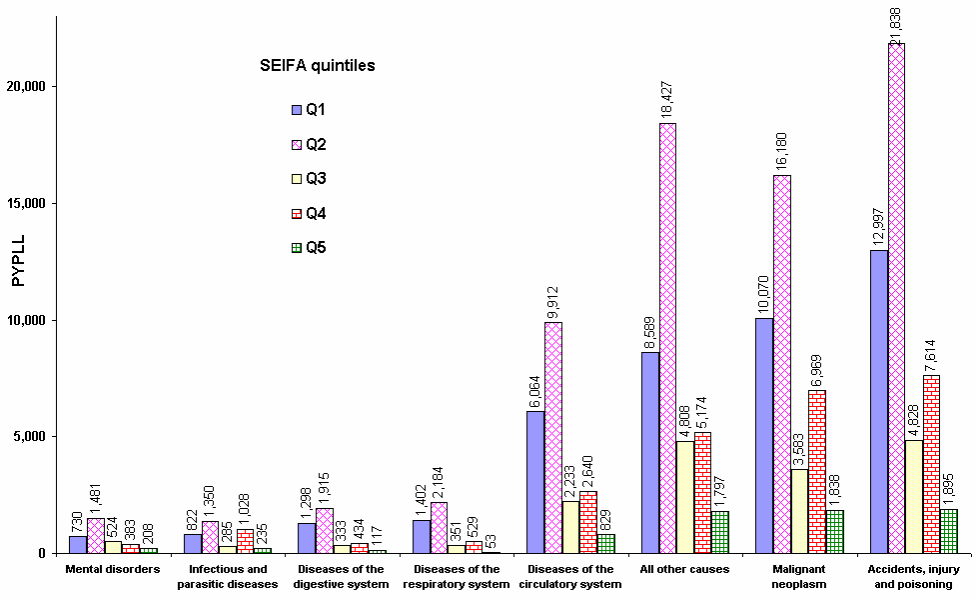
PYPLL of leading causes of death in NSW by the SEIFA based postal area quintiles, 2001.

The distribution of PYPLL by socio-economic status shows that low SEIFA postal areas in NSW had higher mortality in 2001, resulting in higher PYPLLs (Figure 4). The highest burden of premature deaths is due to accidents, injury and poisoning in the bottom two quintiles. The overall disease burden in the bottom two quintiles (115,259 years) is more than twice the PYPLL in the upper three quintiles (49,337 years). The PYPLL as a result of diseases of the cardiovascular system in the bottom two quintiles is about two and a half times higher than the corresponding PYPLLs applying to the upper three quintiles. This discrepancy is highest for diseases of the digestive system (3:1) and lowest for infectious and parasitic diseases (1.4:1). All other deaths (i.e. other than the selected causes of death), which consist mostly of congenital malformations, are also relatively high in low SEIFA postal areas. These data show that an area’s socio-economic status has a high impact on the burden of premature deaths in the community.

Premature deaths caused by accidents, injury and poisoning are responsible for the greatest number of productivity years lost in the working period for both men and women (Figures 5.1 and 5.2). The main causes of death responsible for the positive value of VYPLL in this group of premature deaths for men are: suicide, motor vehicle accidents and accidental drowning; and for women are: suicide, motor vehicle accidents, and motor vehicle accidents involving pedestrians. Of the selected leading causes of death in NSW, the second, and by far less important, is malignant neoplasms. Although VYPLLs turned negative for all causes of death after age 35–39, for accidents, injury and poisoning, VYPLL values did not decrease sharply as for other leading causes of death. The data reveal that the reason for this effect is that the number of deaths caused mainly by diseases of the cardiovascular system and malignant neoplasms increased rapidly after the age 55 while the opposite occurred for deaths from accidents, injury and poisoning.

The crude death rates and the YPLLs calculated using all deaths in NSW show that the leading causes of death are diseases of the cardiovascular system and malignant neoplasms. These two methods emphasize deaths of the elderly, whereas PYPLL and VYPLL methods emphasize the premature deaths of the young. In contrast for VYPLL, malignant neoplasms is ranked last for both men and women. This is because of the negative VYPLLs added to a larger number of men and women who died after the age 45 (i.e. because of negative weights). According to the VYPLL measure, if the cut-off age is set at 45, malignant neoplasms jump to the second rank behind only accidents, injury and poisoning. The differences between these different potential years of life lost measures show that they have inherent limitations.

## 4. Discussion and Conclusions

The main question addressed by this article is whether the economic loss of avoidable death at a younger age is greater than death at old age. There is a wide discrepancy in labour force participation rates by sex with increasing age, with female rates declining more rapidly. While it is possible to argue that, in the context of contemporary NSW, not all economically active persons (i.e. persons aged <15 and persons aged >=65 who are in the labour force) have been taken into account, comparatively the exclusion group is fairly minimal. In a sense PYPLL and VYPLL are generally used to point out avoidable deaths at a younger age and to quantify the mortality.

The rankings of potential life loss measures show that the magnitude of causes of death categories vary according to the method and the cut-off age at death used in the analysis. Since every person is an asset to society, YPLL is a better measure to quantify the total loss to a society due to all deaths. In terms of potential economic loss to society, PYPLL and VYPLL are more appropriate measures, given a justifiable cut-off age. However, there are several inherent limitations in these measures, including: their lack of a standard formula or cut-off age; their dependency on age distribution; their inability to be compared with any other population group; and the fact that they do not measure the cost of death or morbidity associated with specific causes of death. For example, the morbidity and death related costs of a person who dies from a sudden death at age 45 from an accident or myocardial infarction could vary greatly from those of a person’s death at the same age from long-standing malignant neoplasms or disability or lifelong congenital abnormalities with multiple complications, and the latter type of person is less likely to be a ‘producer’ than a ‘consumer’. In other words, medical and social costs associated with one person’s death are likely to be quite different from another’s, depending on the cause and circumstances of the death.

Since the primary sources of data for these measures are the age and causes of death, the validity of the ICD grouping is an important issue. As the data used in this study are based only on the main cause of death, there could be some ICD coding limitations, especially for people who died with multiple complications.

As established in other studies [10, 11] the top ranking loss of productive life to society and the most avoidable cause of death in NSW is the group covering accidents, injury and poisoning. As is the situation in NSW, other studies have found that most deaths caused by accidents, injury and poisoning occur in the younger age groups [12, 13]. The positive weights attributed to these lower ages contribute to positive VYPLL. Most of these preventable deaths with positive VYPLL are concentrated in early adulthood (between age 15 and 24) for both men and women, in contrast to deaths from diseases of the cardiovascular system and malignant neoplasms (with extreme negative VYPLL values at older ages). In order to succeed in further reducing the PYPLL and VYPLL measures, targeted programs are required which aim to reduce avoidable deaths due to accidents, injury and poisoning especially among young adults. Further studies are required to determine which factors contribute to suicide among both young men and women and the most effective remedial actions which can be established within the community. It is well-known that the high death rate among young adults due to motor vehicle accidents is related to speeding, alcohol consumption and drug taking [14, 15]. Therefore driver education, road safety and targeted programs need to be introduced at high school level, as the majority of these deaths occur in the 19–24 years age group.

Looking at PYPLL data in conjunction with the SEIFA index, it is clear that low socio-economic areas have a higher mortality, with the highest burden of premature mortality in these areas being due to accidents, injury and poisoning. According to the data, although Australia (unlike the United States) has a UK style universal health care system, the mortality disparities exist by socio-economic status.

The burden of premature death in the lower SEIFA quintiles is more than twice that for the upper quintiles. Studies have highlighted many disadvantages of living in rural areas in terms of higher disease prevalence and smoking prevalence, and lower occupational status and accessibility of services [16–18]. PYPLL is higher in second lowest quintile than in lowest quintile could be as a result of different age structures or that the persons in the lowest SEIFA quintile postal areas are more likely to be eligible for the government’s low income assistance such as social security payments, government housing and healthcare cards. Those persons a little above the eligibility criteria of the government benefits will most probably be in the second lowest quintile and are likely to be the hardest hit in terms of rising cost of living, services and the rising cost of medicines, even under the pharmaceutical benefit scheme. Therefore further research is required to determine the underlying causes and the remedial actions to reduce the burden of higher premature deaths especially in the second lowest quintile. In order to reduce the rich/poor and urban/rural gap in disease burden due to premature deaths, programs are required to improve the health of people living in lower socio-economic status areas, especially those in rural NSW.

Most health programs are based on national or regional mortality rates and emphasise the prevention of leading causes of death such as diseases of the cardiovascular system and malignant neoplasms. However, a focus on mortality rates tends to lead to programs being weighted on the high deaths among the elderly. This study has revealed that the most years of life lost among the economically active population are due to accidents, injury and poisoning. Therefore attention should also be focused on the reduction of deaths occasioned by motor vehicle accidents, poisoning and suicide. Although this category of deaths cannot be attributed to a particular disease, through education, planning, monitoring and improving safety measures, they are mostly preventable. In order to reduce accidents, injury and poisoning deaths, targeted regional or community level programs are required. Those who are economically active (producers) have a greater responsibility to look after the younger (investors) and the more mature (consumers) members of society.

## Competing interests

The authors declare that they have no competing interests

## Authors’ contributions

DPW designed the study, extracted data from the New South Wales Department of Health compiled deaths database, performed statistical analyses, literature search and written the manuscript. FY and NJP edited the manuscript. All authors read and approved the final manuscript.

## Figures and Tables

**Figure 3.**
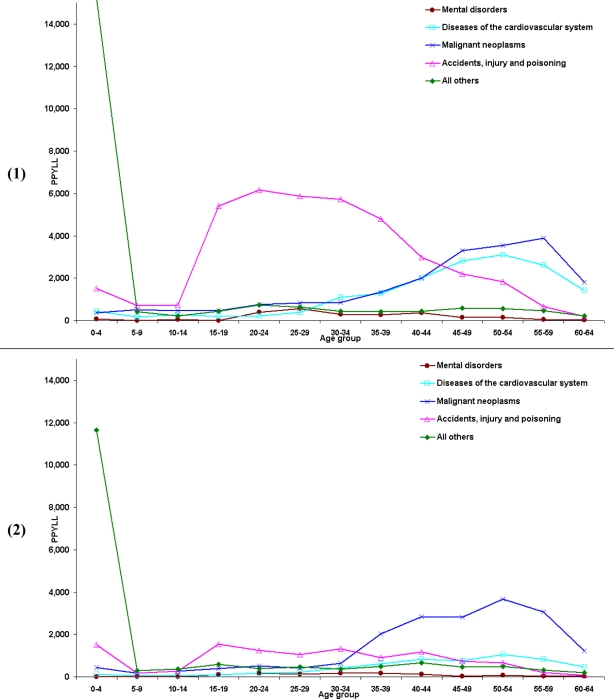
**(1)** Distribution of the contribution of ages to PYPLL by selected causes of death for males in NSW, 2001. **(2)** Distribution of the contribution of ages to PYPLL by selected causes of death for females in NSW, 2001.

**Figure 5.**
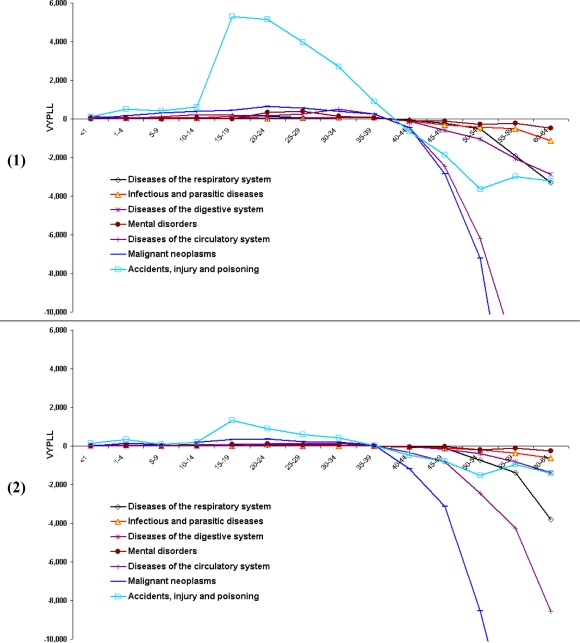
**(1)** Age distribution of VYPLL in NSW for males, 2001. **(2)** Age distribution of VYPLL in NSW for females, 2001.

**Table 1.** Years of potential life lost (YPLL) values computed with different methods and cut-off ages at death for NSW, 2001.

	YPLL-LT1	YPLL-LT2	YPLL-65	YPLL-75	YPLL-85
**Males**	216,319	321,340	107,393	191,242	344,495
**Females**	169,148	238,307	57,203	104,411	202,657
**Persons**	385,467	559,647	164,596	295,653	547,152
**M/F ratio**	1.28	1.35	1.88	1.83	1.70

YPLL-LT1: sum of the differences between the respective life expectancies at birth and age at death, YPLL-LT2: computed using Greville’s method [9] for each five year age group by multiplying the number of deaths by the difference between mean life expectancy in an age and sex group and the mean age at death in the same age sex group, YPLL 65, 75 and 85: represent the cut-off age set at these respective given ages.

## Appendix 1: Formulae used to generate potential life lost measures

Years of potential life lost (YPLL):

$$\text{YPLL}=\sum_{i=0}^{\infty }{\text{d}}_{i}({\text{L}}_{i})$$

Premature years of potential life lost (PYPLL):

$$\text{PYPLL}=\sum_{i=0}^{N}{\text{d}}_{i}(\text{N}-i)$$

Valued years of potential life lost (VYPLL):

$$\text{VYPLL}=\sum_{i=0}^{\infty }{\text{d}}_{i}[\sum_{j=i}^{i+L_{i}}\text{I}(j)]$$

Source for the above formulae:[2], p. 324.

For each year t, the directly standardised PYPLL rate (expressed per 10,000 population) is calculated as follows:

$${\text{PYPLL}}_{\text{t}}=\sum_{i=0}^{\text{N}-1}(\text{N}-\text{i})(\frac{{\text{d}}_{it}}{{\text{p}}_{it}})(\frac{{\text{P}}_{i}}{{\text{P}}_{n}})*10,000$$

Source for the formula: [19], p. 72.

i: age at death, L_i_: life expectancy at age i, N: upper cut of age, W: lower cut off age, I(j): value at age j, d_i_: number of deaths at age i, d_it_: number of deaths at age i in year t, p_it_: number of persons aged i in an area (eg. urban/rural) at time t, P_i_: the number of persons aged i in the standard population, P_n_: the number of persons in the standard population.

## Appendix 2: Information used in the computation of Valued Years of Potential Life Lost

**Table:**

Age at death	Mid-age	Life a expectancy	0–14		15–64		>=65		Net b investment	Potential loss c
			Received	Didn’t receive	Produced	Didn’t produce	Consumed	Didn’t consume		
Males										
<1	0.5	77.1	0.5	14.5	0.0	50.0	0.0	12.6	0.5	23.4
1–4	3.0	76.5	3.0	12.0	0.0	50.0	0.0	14.5	3.0	26.5
5–9	7.5	72.5	7.5	7.5	0.0	50.0	0.0	15.0	7.5	35.0
10–14	12.5	67.6	12.5	2.5	0.0	50.0	0.0	15.1	12.5	44.9
15–19	17.5	62.7	15.0	0.0	2.5	47.5	0.0	15.2	12.5	44.8
20–24	22.5	57.9	15.0	0.0	7.5	42.5	0.0	15.4	7.5	34.6
25–29	27.5	53.1	15.0	0.0	12.5	37.5	0.0	15.6	2.5	24.4
30–34	32.5	48.4	15.0	0.0	17.5	32.5	0.0	15.9	−2.5	14.1
35–39	37.5	43.7	15.0	0.0	22.5	27.5	0.0	16.2	−7.5	3.8
40–44	42.5	39.0	15.0	0.0	27.5	22.5	0.0	16.5	−12.5	−6.5
45–49	47.5	34.3	15.0	0.0	32.5	17.5	0.0	16.8	−17.5	−16.8
50–54	52.5	29.7	15.0	0.0	37.5	12.5	0.0	17.2	−22.5	−27.2
55–59	57.5	25.3	15.0	0.0	42.5	7.5	0.0	17.8	−27.5	−37.8
60–64	62.5	21.0	15.0	0.0	47.5	2.5	0.0	18.5	−32.5	−48.5
Females										
<1	0.5	82.3	0.5	14.5	0.0	50.0	0.0	17.8	0.5	18.2
1–4	3.0	81.7	3.0	12.0	0.0	50.0	0.0	19.7	3.0	21.3
5–9	7.5	77.7	7.5	7.5	0.0	50.0	0.0	20.2	7.5	29.8
10–14	12.5	72.8	12.5	2.5	0.0	50.0	0.0	20.3	12.5	39.7
15–19	17.5	67.8	15.0	0.0	2.5	47.5	0.0	20.3	12.5	39.7
20–24	22.5	62.9	15.0	0.0	7.5	42.5	0.0	20.4	7.5	29.6
25–29	27.5	58.0	15.0	0.0	12.5	37.5	0.0	20.5	2.5	19.5
30–34	32.5	53.1	15.0	0.0	17.5	32.5	0.0	20.6	−2.5	9.4
35–39	37.5	48.2	15.0	0.0	22.5	27.5	0.0	20.7	−7.5	−0.7
40–44	42.5	43.4	15.0	0.0	27.5	22.5	0.0	20.9	−12.5	−10.9
45–49	47.5	38.6	15.0	0.0	32.5	17.5	0.0	21.1	−17.5	−21.1
50–54	52.5	33.9	15.0	0.0	37.5	12.5	0.0	21.4	−22.5	−31.4
55–59	57.5	29.2	15.0	0.0	42.5	7.5	0.0	21.7	−27.5	−41.7
60–64	62.5	24.7	15.0	0.0	47.5	2.5	0.0	22.2	−32.5	−52.2

Three lifetime age segments: investment years (0-14), producer years (15–64), and consumer years (>=65).

a: Life expectancies taken at midpoint age from respective NSW 2000–02 male and females life tables.

b: Net investment = (received) + (consumed) – (produced).

c: Potential loss = (net investment) + (did not produce) – (did not receive) – (did not consume). Note: negative investments and negative losses are gains to society. Source: [2], p. 326
